# *International Classification of Diseases* Coding for Inflammatory Arthritides

**DOI:** 10.1001/jamanetworkopen.2024.6544

**Published:** 2024-04-18

**Authors:** Justin R. Zhu, Anoop R. Galivanche, Michael J. Gouzoulis, Alexander J. Kammien, Stephen M. Gillinov, Rahul H. Jayaram, Jonathan N. Grauer

**Affiliations:** 1 Department of Orthopaedics and Rehabilitation, Yale School of Medicine, New Haven, Connecticut; 2 Department of Orthopaedics, University of California, San Francisco School of Medicine, San Francisco

## Abstract

This quality improvement study investigates usage patterns of codes for inflammatory arthritides under *International Statistical Classification of Diseases and Related Health Problems, Tenth Revision* vs *International Classification of Diseases, Ninth Revision.*

## Introduction

The *International Classification of Diseases* was revised in 2015 from *International Classification of Diseases, Ninth Revision (ICD-9)* to *International Statistical Classification of Diseases and Related Health Problems, Tenth Revision (ICD-10)*, representing the largest revision to the system in 30 years (from 14 500 to 70 000 codes).^1,2,3^ Although *ICD-10* could document conditions with greater granularity, it remains to be seen whether it has been well-utilized up to the 2022 transition to *International Classification of Diseases, 11th Revision (ICD-11)*.

With *ICD-10*, the number of inflammatory arthritis codes increased from 14 to 425. Although this allows for better characterization of such conditions for more than 1.3 million affected US individuals,^4^ the greater granularity is useful only if the breadth of codes is utilized. The purpose of this quality improvement study was to investigate usage patterns of inflammatory arthritides codes under *ICD-10* vs *ICD-9*.

## Methods

M151Ortho is a national multi-insurance administrative claims data set by Pearldiver Technologies that was used to identify patients with inflammatory arthritides with *ICD-9* and *ICD-10* codes. Because Pearldiver data are deidentified, the Yale University institutional review board deemed that this quality improvement study did not meet the federal definition of research and did not require approval or informed consent, in accordance with 45 CFR §46. This report follows STROBE reporting guidelines. *ICD-9* inflammatory arthritis codes include 714.0X to 714.9X (14 codes). *ICD-10* codes include M05.XXX, M06.XXX, M08.XXX, and M12.00X-M12.09X (438 codes). If a patient had multiple encounters, the earliest encounter was used.

Patients with *ICD-10* codes were grouped by year from 2015 to 2021 and by clinician specialty code. Clinicians were categorized as primary care physicians, rheumatologists, orthopedic surgeons, and other physicians.

Distributions of code utilization were compared between year-by-year subgroups and clinician-by-clinician subgroups. χ^2^ and 2-sample Kolmogorov tests were performed using Bellwether software version 2.1 (Pearldiver) and R software version 4.3.0 (R Project for Statistical Computing). Significance was set at 2-tailed α = .05.

## Results

In total, 5 152 250 patients (3 817 817 female [74.1%]; mean [SD] age, 58.2 [16.4] years) with inflammatory arthritides were identified; 2 626 584 (51.0%) were coded in *ICD-9* and 2 525 666 (49.0%) in *ICD-10*. For *ICD-9*, 4 of 14 inflammatory arthritides codes (28.6% of available codes) were higher-usage codes (used >1% of the time). For *ICD-10*, 9 of 438 codes (2.1%) were higher-usage (Table). Of the top 20 codes, 65% contained *unspecified* or *other specified* in the verbiage.

**Table.** Top 10 *ICD-9* and *ICD-10* Codes for Inflammatory Arthritides by Usage

Rank	*ICD-9* (n = 2 626 584 patients)		*ICD-10* (n = 2 525 666 patients)	
	Code name	Patients, No. (%)	Code name	Patients, No. (%)
1	714.0 Rheumatoid arthritis	2 000 071 (76.1)	M06.9 Rheumatoid arthritis unspecified	1 340 662 (53.1)
2	714.9 Unspecified inflammatory polyarthropathy	500 424 (19.1)	M06.4 Inflammatory polyarthropathy	394 214 (15.6)
3	714.89 Other specified inflammatory polyarthropathies	47 208 (1.8)	M05.79 Rheumatoid arthritis with rheumatoid factor of multiple sites without organ or systems involvement	179 450 (7.1)
4	714.30 Polyarticular juvenile rheumatoid arthritis chronic or unspecified	38 750 (1.5)	M06.09 Rheumatoid arthritis without rheumatoid factor multiple sites	112 237 (4.4)
5	714.2 Other rheumatoid arthritis with visceral or systemic involvement	12 242 (0.5)	M05.9 Rheumatoid arthritis with rheumatoid factor unspecified	84 559 (3.3)
6	714.4 Chronic postrheumatic arthropathy	6445 (0.3)	M06.00 Rheumatoid arthritis without rheumatoid factor unspecified site	43 930 (1.7)
7	714.32 Pauciarticular juvenile rheumatoid arthritis	5423 (0.2)	M05.89 Other rheumatoid arthritis with rheumatoid factor of multiple sites	39 655 (1.6)
8	714.1 Felty’s syndrome	4811 (0.2)	M06.89 Other specified rheumatoid arthritis multiple sites	35 713 (1.4)
9	714.81 Rheumatoid lung	4398 (0.2)	M05.40 Rheumatoid myopathy with rheumatoid arthritis of unspecified site	27 114 (1.1)
10	714.31 Polyarticular juvenile rheumatoid arthritis acute	1824 (0.0)	M08.00 Unspecified juvenile arthritis of unspecified site	19 551 (0.8)

Abbreviations: *ICD-9*, *International Classification of Diseases, Ninth Revision*;* ICD-10*,* International Statistical Classification of Diseases and Related Health Problems, Tenth Revision.*

To assess for a learning curve in *ICD-10* usage, year-by-year subgroups were tabulated for number and percentage of higher-usage codes (Figure). There was no significant change from year to year.

**Figure.**
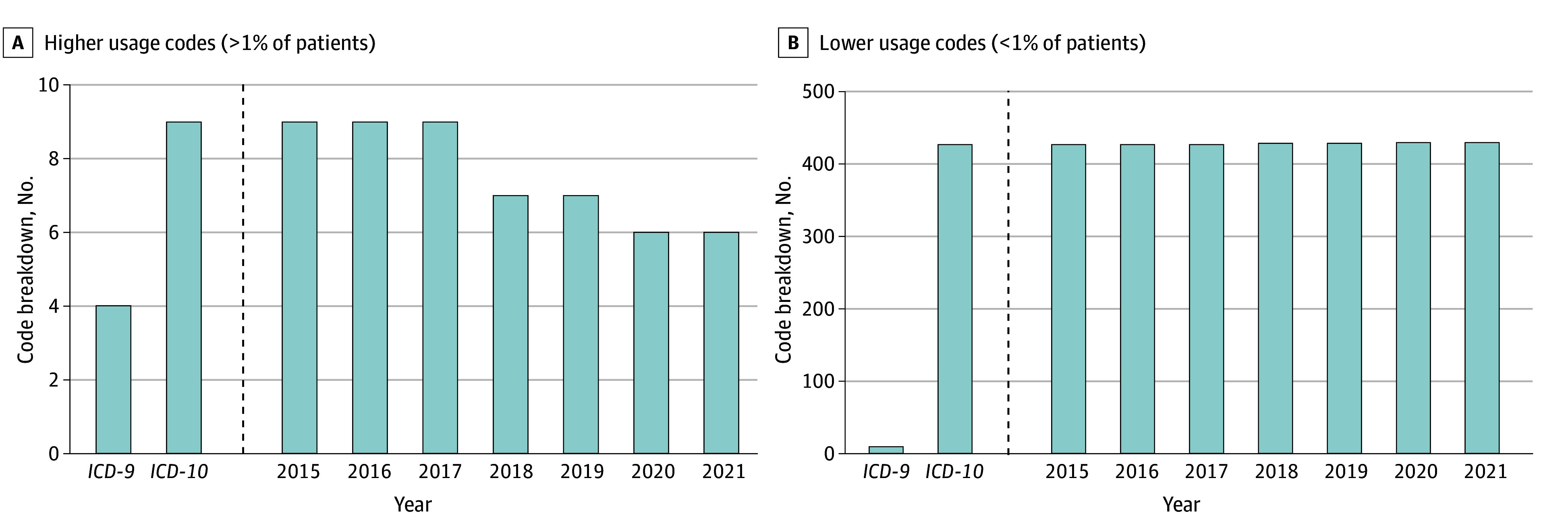
Higher vs Lower Usage *International Classification of Diseases, Ninth Revision (ICD-9)* and *International Statistical Classification of Diseases and Related Health Problems, Tenth Revision (ICD-10)* Codes Graphs show higher usage codes, defined as those accounting for >1% (A), and lower usage codes, defined as those accounting for <1% of patients (B), presented in total and by year following the introduction of *ICD-10*. In panel A, there were 9 codes (2.1%) each for 2015, 2016, and 2017; 7 (1.6%) each for 2018 and 2019; and 6 (1.4%) each for 2020 and 2021. There was no statistically significant change in utilization of higher-usage codes from one year to the next following the introduction of *ICD-10* (*P* = .94).

To assess usage of *ICD-10* across specialties, clinician subgroups were assessed for number of higher-usage codes: 9 codes (2.1%) for primary care, 8 (2.1%) for rheumatology, 2 (0.5%) for orthopedics, and 6 (1.4%) for other specialties. There were no differences between primary care physicians, rheumatologists, and other physicians, whereas orthopedists used significantly fewer codes.

## Discussion

The transition to *ICD-10* afforded the potential of increased granularity of inflammatory arthritides diagnosis codes for clinical care, research, and billing purposes. This quality improvement study found that, despite a 30-fold increase in codes, adoption of *ICD-10* has been poor; only 2.1% of available codes were used more than 1% of the time and were nonspecific. This may reflect the lack of financial incentive for accurate *ICD-10* coding, because *ICD-10* is not tied to reimbursement.

When assessed by year, the percentage of codes used with regularity did not increase. When assessed by clinician specialty, the percentage of codes used was not better for rheumatologists (who might be expected to be more refined users of such codes) than primary care clinicians. This study is limited by potential coding inaccuracies and cannot establish causality.

Overall, consistent with findings in other areas,^5,6^ the data suggest room for better application of inflammatory arthritis codes. This will be important in years to come if the benefits of* ICD* expansion are to be realized.

## Notes

Supplement.
Data Sharing Statement
