# Supplementary material for: International Classification of Diseases Coding for Inflammatory Arthritides
Source: JAMA Netw Open. 2024 Apr 18;7(4):e246544. doi: 10.1001/jamanetworkopen.2024.6544 (PMC12124733; doi:10.1001/jamanetworkopen.2024.6544)
Supplement: Supplement. — Data Sharing Statement [file jamanetwopen-e246544-s001.pdf]

Data Sharing Statement

Zhu. International Classification of Diseases Coding for Inflammatory Arthritides. *JAMA Netw Open*. Published April 18, 2024. doi:10.1001/jamanetworkopen.2024.6544

Data
Data available: No

Additional Information
Explanation for why data not available: Patient output from national dataset in aggregated form and individual patient data not available for sharing
